# Tunable resonance transmission modes in hybrid heterostructures based on porous silicon

**DOI:** 10.1186/1556-276X-7-392

**Published:** 2012-07-13

**Authors:** Karina S Pérez, J Octavio Estevez, Antonio Méndez-Blas, Jesús Arriaga, Gabriela Palestino, Miguel E Mora-Ramos

**Affiliations:** 1 Instituto de Física, Universidad Autónoma de Puebla, A.P. J-48, Puebla, 72570, México; 2 Facultad de Ciencias Químicas, Universidad Autónoma de San Luis Potosí, Alvaro Obregón 64, San Luis Potosí, 78000, México; 3 Facultad de Ciencias, Universidad Autónoma del Estado de Morelos, Av. Universidad 1001, Cuernavaca, CP 62209, Morelos, México

**Keywords:** Fibonacci substructure, Porous silicon, Heterostructures

## Abstract

In this work, we report the experimental results and theoretical analysis of strong localization of resonance transmission modes generated by hybrid periodic/quasiperiodic heterostructures (HHs) based on porous silicon. The HHs are formed by stacking a quasiperiodic Fibonacci (FN) substructure between two distributed Bragg reflectors (DBRs). FN substructure defines the number of strong localized modes that can be tunable at any given wavelength and be unfolded when a partial periodicity condition is imposed. These structures show interesting properties for biomaterials research, biosensor applications and basic studies of adsorption of organic molecules. We also demonstrate the sensitivity of HHs to material infiltration.

## Background

Photonic crystals are attractive optical materials to control and manipulate the flow of light. A periodic dielectric system (multilayered), typically consisting of two alternated dielectric materials with periodic variation of refractive index (*n*), is the simplest photonic crystal (PC) [1]. The propagation of electromagnetic radiation in PCs is forbidden in specific wavelength ranges (photonic band gaps or PBGs) because the light wave is scattered at the layers’ interfaces, so the multiple-scattered waves interfere destructively into the material [2]. The behavior of light in a periodic scattering media can be described by Bloch states [3]. In addition, localized modes can appear into the PBGs by breaking the periodicity of the dielectric multilayer, i.e., by introducing a defect into a PC [4] that allows a narrow range of light wave frequencies to propagate through the whole structure. Physically, the defect is a single layer with different optical parameters (refractive index or thickness) or a completely different multilayer substructure [5]. Novel applications to optical devices, such as all-optical circuit, dielectric mirrors, Fabry-Perot filters, distributed feedback lasers, etc., have been proposed for the above-mentioned structures with localized modes. However, not only PCs based on periodic or periodical structures with defects are of interest but also deterministic aperiodic systems or quasicrystals because of their unexpected optical features [6-10]. The quasicrystals can be considered as a class of complex dielectric structures between ordered crystals and fully random structures. These structures show PBGs, but they are non-periodic multilayer structures. The quasicrystal structures are formed of layers with optical parameters that obey deterministic rules [11]. The Fibonacci and Thue-Morse mathematical sequences are two examples of numbers generated by deterministic rules. In order to associate these kinds of sequences to multilayer structures, it is necessary to define the so-called generators, i.e., initial layers with specific values of their optical properties.

Important applications for quasicrystal-type structures, such as band-edge lasing [12], optical frequency-selective filters [13], efficient nonlinear filters [14], bistability [15], and switching [16], have been suggested. In optical sensor applications based on PCs, the sensitivity is associated with the capacity for binding analyte molecules to the surface of the layers [17]. Porous silicon (PSi) has a great capacity of binding molecules at its surface due to its large specific superficial area (≥ 200 m^2^/cm^3^) [18]. The biocompatibility of the PSi [19] makes it a promising material to be used as a biosensor. PSi is a nanostructured material [20] considered as a mix of silicon and air with effective optical parameters, and its optical and structural features allow the fabrication of complex PCs [21-23]. Since PSi is obtained by electrochemical etching, and the porosity is directly related to the refractive index [24], it is possible to control its optical parameters by controlling the thickness and porosity by means of time and applied current during the process, respectively [25]. These features allow the fabrication of several types of one-dimensional (1D) PCs and the introduction of complex defect layers into a periodic multilayer structure. The strong confinement of electromagnetic fields within the engineered defect layers is an advantage offered by PCs because it is highly dependent on the refractive indices and thickness of each constituent layer; any change in these parameters is reflected as a change in the optical response. It is possible to achieve a spectral shift of the localized modes when a slight change of refractive index in some layers or on the whole structure is induced. Such displacements could be obtained by natural or thermal oxidation of the PSi structure or by introducing into the pores some specific substances. This advantage can be exploited particularly in biosensing applications due to its high sensitivity requirements compared to other sensors, which only use the weak evanescent field for sensing [26]. It is possible to obtain small spectral shifts in the reflectance or transmittance measures by introducing solutions or analytes into the pores of PSi, which can be monitored with exceptional precision. Numerous works have been published based on this idea, but only the simplest PSi structures (i. e. monolayers, distributed Bragg reflectors (DBRs), and several types of filters) have been used to study different molecular species as proteins [27,28], DNA [29], solvents [30], neurons [31], etc. However, the optical features of PSi complex multilayer structures have not been explored widely for their application in the biosensing area. From this perspective, our interest lies on the fabrication of a highly efficient photonic structure for biosensing purposes. To achieve this goal, in this work, the fabrication of hybrid heterostructures (HHs) based on PSi is proposed. The HHs are a complex combination of the features of periodic and quasiperiodic photonic structures. The study of hybrid heterostructures has been approached in previous works by other authors but only in the theoretical aspect, and they not consider PSi nanostructures [32,33]. It is the first experimental study of HHs based on Fibonacci (FN) sequences.

## Methods

The HHs are formed by stacking a FN substructure between two DBRs in the sequence (DBR)^*N*^−(FN)^*M*^−(DBR)^*N*^[34]. The DBRs are formed by a periodic arrangement of two alternated layers, *A* and *BN* times. The FN sequences are generated by the recursion relation *F*_*M*_=*F*_*M*−1_ + *F*_*M*−2_where *M* represents the order of the sequence ( *M*=2,3,4,…). It is possible to generate dielectric multilayered structures that follow the FN mathematical sequence of any order by choosing *F*_0_=*C* and *F*_1_=*CD* where *C* and *D* are two different layers. For example, *F*_2_=*CDCF*_3_=*CDCCDF*_4_=*CDCCDCDC*, and so on. The HHs present strongly localized transmission modes as a function of the design parameters and can be localized over a wide range of frequencies. The HHs based on PSi studied in this work were obtained by electrochemical etching from boron-doped silicon wafers (100)-oriented and 0.007 to 0.013 *Ω* cm resistivity. A small piece of silicon wafer was used as substrate for etching in an HF (40%) and ethanol (99.98%) solution in a volumetric ratio of 1:1. More details about the process can be found in reference [5]. In order to calculate the refractive index of each layer for a given current density, we use the effective medium approximation of the Bruggeman’s model [35]:

(1) $$f_{P}\left(\frac{\varepsilon_{\mathrm{Si}}-\varepsilon_{\mathrm{PSi}}}{\varepsilon_{\mathrm{Si}}+2\varepsilon_{\mathrm{PSi}}}\right)+(1-f_{P})\left(\frac{1-\varepsilon_{\mathrm{PSi}}}{1+2\varepsilon_{\mathrm{PSi}}}\right)=0.$$

This model provides the complex dielectric constant of PSi (*ε*_PSi_) as a function of the dielectric constants of silicon (*ε*_Si_) and air (*ε*_air_), as well as porosity *f*_p_. The values of *f*_p_were calculated by the gravimetrical method. As the PSi layers consist of only two optical media, the *ε*_PSi_ value is intermediate between the *ε*_Si_ and *ε*_air_ values, weighed by the volume fraction 1−*f*_p_and *f*_p_, respectively, (in Equation 1 we take *ε*_air_=1). Solving for *ε*_PSi_in Equation 1, we obtain

(2) $$\begin{array}{cc} \varepsilon_{\mathrm{PSi}} & =\frac{1}{4}\left\{\left(2-\varepsilon_{\mathrm{Si}})+3f_{P}(\varepsilon_{\mathrm{Si}}-1\right)\right)\\ \left(\;+{\left[{\left((2-\varepsilon_{\mathrm{Si}})+3f_{P}(\varepsilon_{\mathrm{Si}}-1)\right)}^{2}+8\varepsilon_{\mathrm{Si}}\right]}^{1/2}\right\}. \end{array}$$

Cauchy model is useful to know the refractive index (*n*) and the extinction coefficient ( *k*) for dielectric materials (with exponential absorption), far from the absorption bands [36], by the equations

(3) $$n(\lambda )=a+\frac{b}{\lambda^{2}}+\frac{c}{\lambda^{4}}$$

(4) $$k(\lambda )=d\exp\left(\frac{e}{\lambda }\right).$$

**Figure 1:**
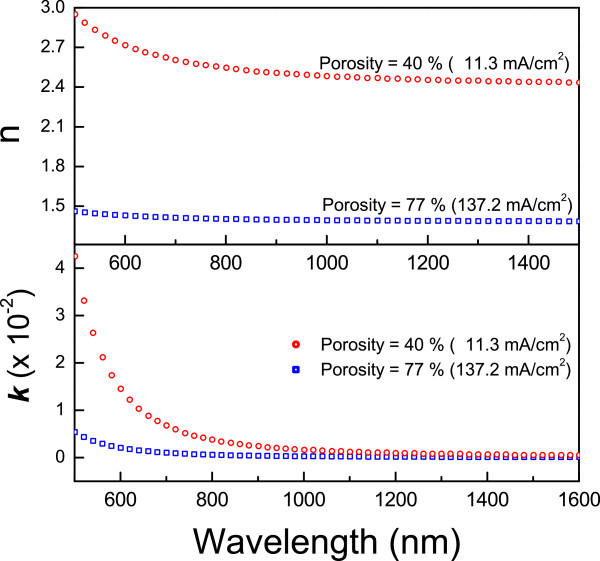
**Optical parameters*n*and*k*vs wavelength.** The values for layers with low and high porosity (40% and 77%, respectively) were calculated using the Bruggeman and Cauchy models.

Note that this model is defined by five parameters: *abcd*, and *e*. These parameters are adjusted to experimental values of *n* and *k* for crystalline silicon from reference [37]. For example, in Figure 1*n* and *k* are plotted for two different values of porosity (low and high) which correspond to the design parameters for the *A* and *B* layers (40% and 77% of porosity, respectively) of the DBRs mentioned above. In all the simulations, we consider Equations 3 and 4.

**Figure 2:**
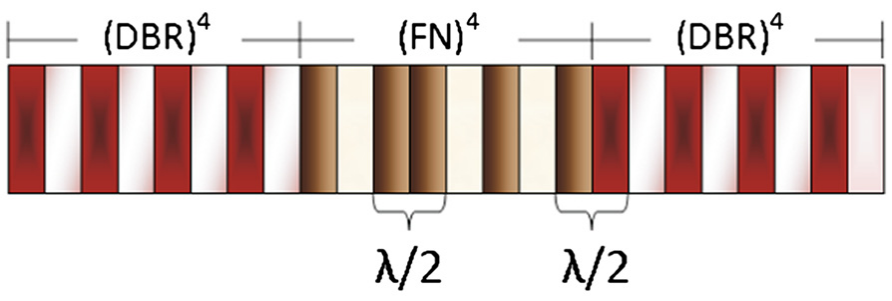
**Schematic of the HHs.** The number of periods for DBRs is *N* = 4, and the order of the Fibonacci substructure is *M* = 4. Resonances will be observed in reflectance spectra due to the *λ*/2 layer defects.

For a *λ* value around 1.0 *μ*m, the refractive index and thickness for layer *A* is 1.4 and 178.5 nm, and for layer *B*, 2.5 and 100 nm, respectively. The refractive indices for *C* and *D* layers are 1.6 and 2.2, and their thicknesses are 156.25 and 113.64 nm, respectively. In all these layers, the optical thickness *nd*, where *n* is the refractive index and *d* is the physical thickness, has the constant value *λ*/4 = 250 nm. A schematic of the structure with the sequence (DBR)^4^−(FN)^4^−(DBR)^4^is shown in Figure 2, in which it is possible to observe the formation of two optical modes corresponding to *λ*/2 defects.

On the basis of this idea, structures with different order of the FN substructure were designed to show resonances in the IR region. These resonances can be seen experimentally like narrow transmission bands in a reflectance spectrum. When the FN substructure is third, fourth, or fifth order (*M* = 3, 4, 5…), it presents one, two, or three resonance transmission modes, respectively. The number of resonances is in direct analogy with the FN sequence (see Table 1). *N* = 4 in DBRs substructures was kept constant for all the structures.

**Table 1:** Fibonacci order, substructure, and sequence


	**FN substructure**	**FN sequence and number**
	**Generators:**	**of defects Generators:**
**Order**	***F***_**0**_**= C,*F***_**1**_**= CD**	***F***_**0**_**= 0,*F***_**1**_**= 1**
2	*CDC*	1
3	*CDCCD*	1
4	*CDCCDCDC*	2
5	*CDCCDCDCCDCCD*	3
6	*CDCCDCDCCDCCDCDCCDCDC*	5
7	*CDCCDCDCCDCCDCDCCDCDC*…	8

FN, Fibonacci. *F*_0_and *F*_1_are the initial layers/values in the recursion relation.

The reflectivity measurements of 1D photonic crystals based on PSi structures were carried out in an Agilent spectrophotometer (Cary 5000 UV-VIS-NIR, Agilent Technologies, Santa Clara, CA, USA), with the specular reflectance accessory (VASRA). All the spectra were measured at an angle of incidence of 20°. Reflectivity measurements were carried out with a p-polarized beam. The experimental results were compared with those given by the theory.

### Theoretical model

To model the propagation of light in these systems, we used the transfer matrix method [38]. If we consider an electromagnetic (EM) wave propagating in the structure with propagation constant **k**= **k**_∥_ + **k**_*z*_, there are two independent EM modes: transverse-magnetic (TM) modes and transverse electric (TE) modes. The electric (magnetic) field for the TE (TM) mode is perpendicular to the plane defined by the wave vector and the direction of periodicity. Using the transfer matrix formalism, we can relate the amplitudes of the fields $E_{\mathrm{j\mu}}^{+}$ and $\left(E_{\mathrm{j\mu}}^{-}\right)$ in the *j*− *th* layer of the system to the amplitudes of the field in the ( *j* + 1)− *th*layer according to

(5) $$\left(\begin{array}{c} E_{\mathrm{j\mu}}^{+}\\ E_{\mathrm{j\mu}}^{-} \end{array}\right)=M_{\mu }\left(\begin{array}{c} E_{j+1\mu }^{+}\\ E_{j+1\mu }^{-} \end{array}\right)$$

where $E_{\mathrm{j\mu}}^{+}(E_{\mathrm{j\mu}}^{-})$ is the amplitude of the wave in the layer *j*, with polarization *μ*( *μ* = *sp*) traveling to the right (left). For the case considered in this work, the total transfer matrix of the system can be written as a product of matrices of the type [39]

(6) $$M_{\mathrm{j\mu}}=\left(\begin{array}{c} \;\;\;\cos(\phi_{j})\;-i\sin(\phi_{j})/q_{\mathrm{j\mu}}\\ -iq_{\mathrm{j\mu}}\sin(\phi_{j})\;\;\;\cos(\phi_{j}) \end{array}\right)$$

where $q_{\mathrm{j\mu}}=k_{j}^{2}/k_{\mathrm{jz}}$ for *p*-polarization and *q*_*jμ*_=(*k*_*jz*_) for *s*-polarization; *ϕ*_*j*_ = *k*_*jz*_*d*_*j*_*k*_*j*_ = (*ω*/ *c*) *n*_*j*_*k*_*jz*_is the component of the wave vector along the growth direction of the system in the *j*− *th* layer given by $k_{\mathrm{jz}}\;=\;\sqrt{k_{j}^{2}-k_{∥}^{2}}$; and $n_{j}\;=\;\sqrt{\varepsilon_{j}}$ is the complex refractive index. The reflectivity of the system is given in terms of the matrix elements of the total transfer matrix according to $R={\left|M_{21}/M_{11}\right|}^{2}$. We have implemented a realistic transfer matrix approach by considering the wavelength dependence of the refractive index as well as the optical absorption. Absorption is a very important parameter, especially when considering the visible region of the electromagnetic spectrum.

## Results and discussion

**Figure 3:**
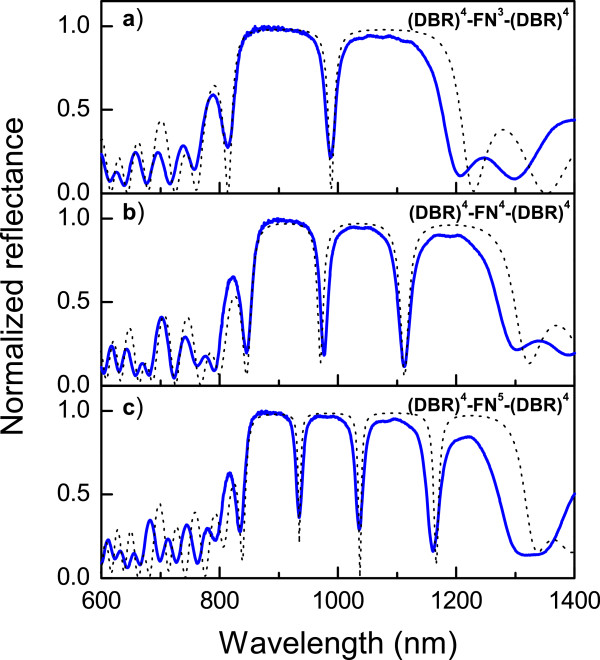
**Reflectance measurements of the HHs and their theoretical simulation.** The FN substructure was **(a)** third order, **(b)** fourth order, and **(c)** fifth order.

In Figure 3, we present the optical reflectivity measurements of three HHs (solid line). In all cases, strongly localized transmission modes can be seen. The third-order FN substructure (Figure 3a) (DBR)^4^−(FN)^3^−(DBR)^4^ presents one localized transmission mode at 988 nm with 19.7 nm of full width at half-maximum (FWHM). The localized mode is produced by the two adjacent *λ*/4 layers, in this case, two *C* layers in the FN substructure. The HH based on the fourth-order FN substructure (Figure 3b) presents two localized modes at 977 and 1,112.4 nm with a FWHM of 15.7 and 22.7 nm, respectively. The first localized transmission mode is produced again by two *C* layers in the FN substructure. However, the second defect is produced by a *C* layer from an FN substructure adjacent to an *A* layer from the DBR substructure. Even though the refractive index and the thickness for both layers are different, their optical thickness is the same ( *nd*= *λ*/4), so the *λ*/2 condition is kept. Furthermore, in this case, the condition of periodicity is met before and after the defect. In Figure 3c, the fifth order of the FN substructure between the DBRs produces three resonant modes at 934.5, 1,036.3, and 1,160.8 nm with FWHMs of 13.2, 14.8, and 23.6, respectively. In this case, all the localized modes are due to three pairs of *C* layers in the FN substructure. For HHs corresponding to the upper order of FN substructures, similar defects are found, and the number of defects follows the numerical Fibonacci’s sequence (see Table 1). The optical modes can be designed to appear at almost any wavelength because they depend on the optical thickness, i.e., we can design specific PSi structures with the correct refractive indices and thicknesses to match any electromagnetic region. Theoretical simulations of the same HHs are also plotted in Figure 3 (dotted line). In these simulations, we took the values of refractive index and thickness mentioned in the ‘Methods’ section but considering ±0.05 deviation in the refractive index values. From this figure, the excellent agreement with the experimental results can be seen, which gives us certainty on the theoretical modeling. This model takes into account the *n*( *λ*) and *k*( *λ*) dependence for each layer.

**Figure 4:**
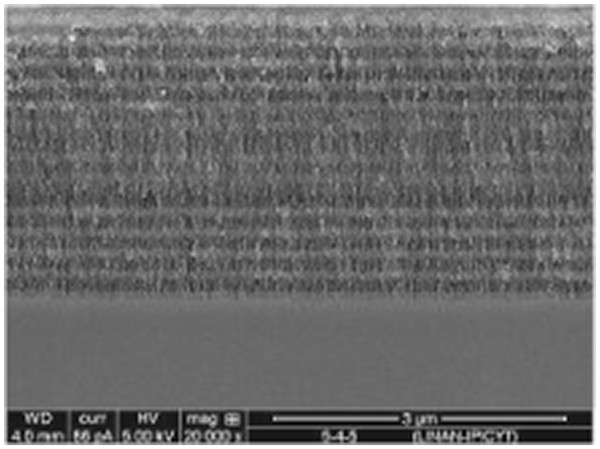
**HRSEM of a HHs with the sequence(DBR)**^**5**^**−(FN)**^**4**^**−(DBR)**^**5**^. Dark and clear layers correspond to high and low porosities, respectively.

Figure 4 shows a high-resolution scanning electron microscopy (HRSEM) image of a HH with a FN substructure of fourth order; in this particular sample, there are five periods for the DBRs. Three zones can be seen clearly: the top and bottom zones correspond to DBRs, and the middle one corresponds to the FN substructure. The dark and clear zones are due to layers with high (low) and low (high) porosities (refractive index value), respectively. The lower contrast in the layers of FN substructure is due to the low contrast in the refractive index of their constituent layers compared to DBR layers. Measures of the thickness of the layer’s in this and other HRSEM images showed an excellent agreement between the observed thickness values and those calculated by the gravimetrical procedure for each layer.

**Figure 5:**
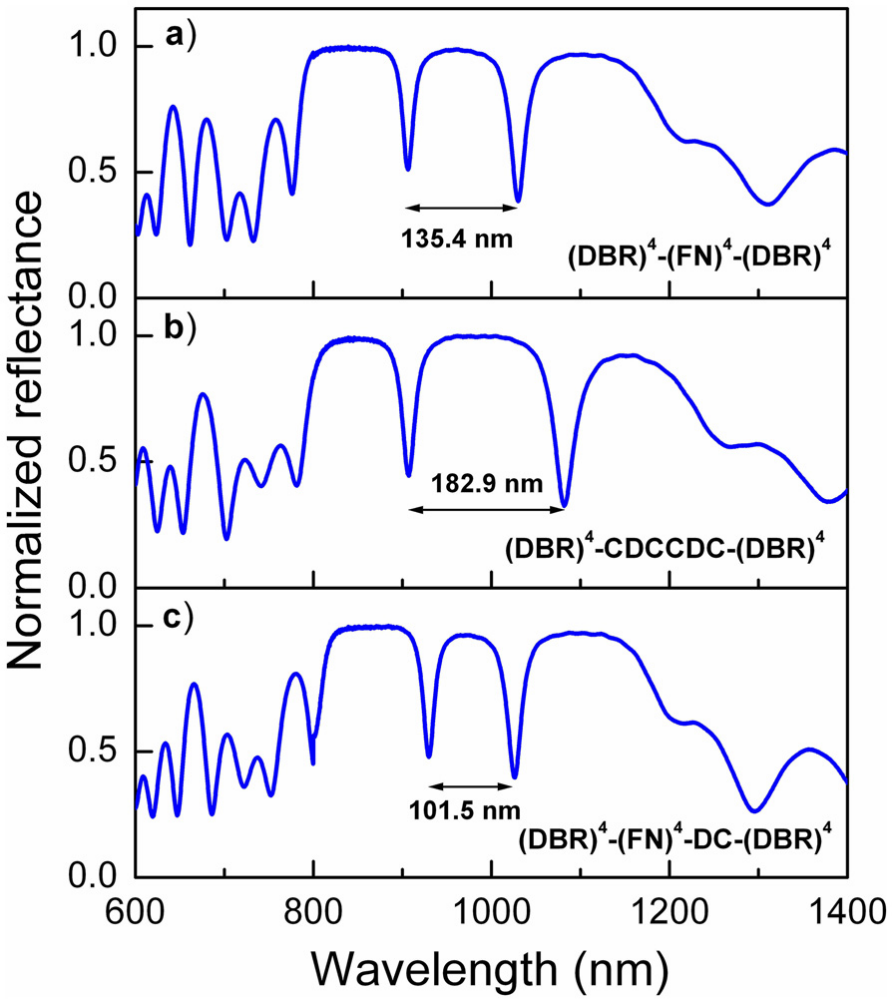
**Reflectance of modified HHs in the FN substructure.(a)** Reference structure. **(b)** The last two layers of the FN substructure have been removed. **(c)** A pair of DC layers has been added to the end of the FN substructure.

In order to show the effect of two specific modifications on the HHs, we chose the structure (DBR)^4^−(FN)^4^−(DBR)^4^ shown in Figure 3b. The first modification consists of adding or removing layers from the FN substructure; in any case, the Fibonacci sequence is lost. However, the spectral positions where the resonances appear can be changed by this modification. In Figure 5, the effect of removing some layers from the FN substructure can be observed. The original structure from Figure 3b is shown again in Figure 5a for reference. From the (DBR)^4^−(FN)^4^−(DBR)^4^structure, the two last layers from the FN substructure were removed, and the resulting structure is now (DBR)^4^−(CDCCDC)−(DBR)^4^. In Figure 5b, it can be seen that the two resonances are localized at 960.7 and 1,143.6 nm with FWHMs of 17.4 and 28.6, respectively, so the range between the resonances has increased to 182.9 nm, the range being 135.4 nm before modifying the FN sequence (Figure 5a). Moreover, the addition of a pair of DC layers to the end of the FN substructure results in the structure (DBR)^4^−(FN)^4^DC−(DBR)^4^. Figure 5c shows the resulting reflectance spectra after the last modification. Now, the optical resonances appear at 1,013.9 and 1,118.4 nm, so the new range between the two resonances is 101.5 nm, i.e., the difference of the two resonances’ spectral position has decreased, compared to original HHs. This result can be explained taking into account the interaction between the defect modes. The larger the physical distance between the defect layers, the weaker is the interaction of the eigenmodes so that they can appear at the nearest frequencies when there is a very long distance between defects layers. On the other hand, when the distance between defects is short, the interaction of the eigenmodes increases, but they cannot appear at the same energy or frequency in the optical spectra. Consequently, the localized modes in the reflectance spectra appear more separated [40]. These important features prove the flexibility of the structures and their capacity to localize the resonant modes at almost any wavelength as a function of the design parameters. This tunability can be exploited in optical devices, particularly in the biosensing area.

**Figure 6:**
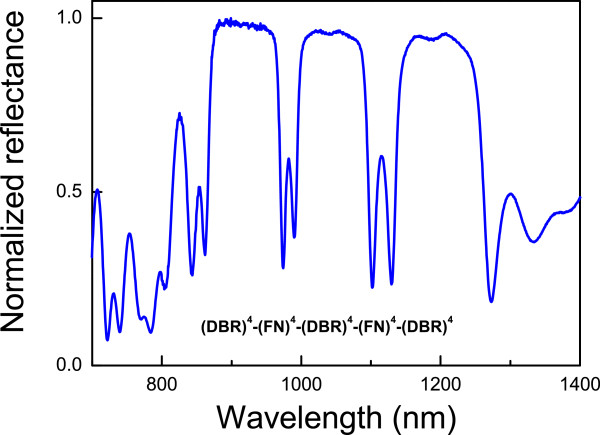
**Reflectance spectra of HHs with a unit cell repeated twice.** The structure has two resonant modes unfolded.

The second modification to the structure was carried out by imposing a partial periodicity on the structure. The unit cell is now formed by a DBR and a FN substructure. In order to observe the split of the resonances, a DBR substructure was added to the end of the whole structure. In this way, FN substructures are always seen as defects. Applying this modification to the structure and using the appropriated optical parameters, it is possible to observe the unfolded resonance mode. The number of splitting modes from each resonance modes depends on the number of times that the unit cell is repeated in the whole structure. For example, repeating two times the unit cell in the structure (DBR)^4^−(FN)^4^−(DBR)^4^, we obtain (DBR)^4^−(FN)^4^−(DBR)^4^−(FN)^4^−(DBR)^4^, and the resulting reflectance spectra of the new structure (see Figure 6) show twofold of each resonance mode that appears in the original structure in Figure 3b. A threefold splitting can be obtained by repeating three times the unit cell; fourfold splitting would correspond to four times, and so on.

The effective refractive index in PSi is the result of a homogeneous mixture of air and silicon, so any material infiltration into the pores displaces off part of the air. Consequently, a red- or blueshift could be expected in the optical reflectance spectra due to a partial change of the optical parameters. The same effect is expected in monolayers and multilayers or even in more complex PSi structures. So, red- or blueshift can be monitored in order to estimate the sensitivity of the structures. To achieve this in multilayered structures, it is preferable to have strong localized modes in order to follow more easily the spectral displacements. The HHs developed in this work have the strong localized modes needed, but they are observed in very complex structures. However, we want to demonstrate that these complex structures make them more sensitive to material infiltration, in particular, when biological molecules are placed into the pores of HHs based on PSi. In order to know the feasibility of the HHs to be used as biosensors, 3-aminopropyltriethoxysilane (APTES) molecule was attached to the internal surface of the structure [41]. To do this, it is necessary to follow a specific process described here: (1) HHs based on PSi were thermally stabilized at 900°C under oxygen flow; (2) APTES silanization was done in a 5% solution with toluene during 1.5 h; (3) the samples were rinsed with toluene and dried under nitrogen flow, and finally, (4) samples were baked in an oven at 110°C for 15 min. The procedure to silane’s modification is well described elsewhere [42].

**Figure 7:**
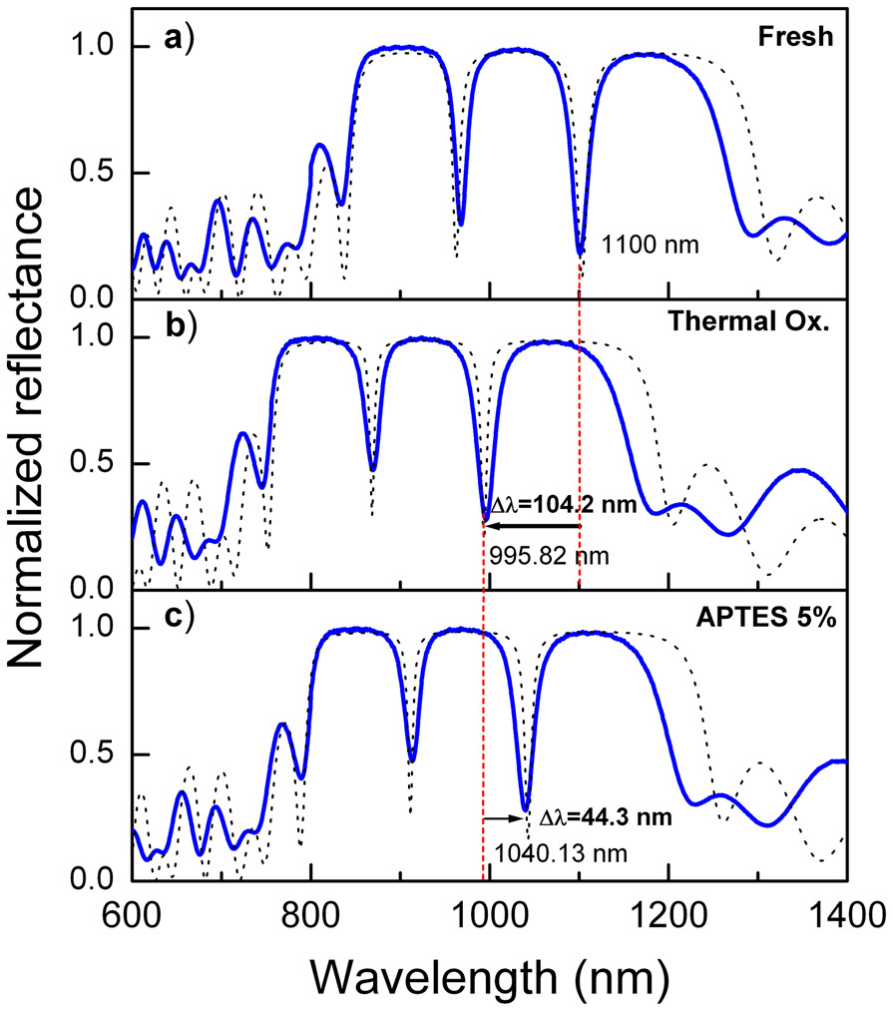
**Experimental reflectance data of the (DBR)**^**4**^**−(FN)**^**4**^**−(DBR)**^**4**^**HHs.** Solid line: **(a)** As obtained , **(b)** thermally oxidized at 900°C, and **(c)** after APTES infiltration. Dashed line, theoretical simulation.

In Figure 7, the experimental reflectance spectra for the infiltration process can be observed (solid line). A blue- or redshift of the (DBR)^4^−(FN)^4^−(DBR)^4^HHs in the three stages of the procedure can be seen clearly. Figure 7a shows the reflectance spectra of the sample as prepared. The thermal stabilization of the samples in oxygen atmosphere produces a partial transformation of the silicon filaments to silicon dioxide. The refractive index of silicon dioxide is lower with respect to silicon; therefore, a decrease in the effective refractive index of the layers [43] in the HHs can be detected by a large displacement of the resonant modes to short wavelengths. This blueshift is observed in Figure 7b and compared with a sample as prepared in Figure 7a. Moreover, the infiltration of biological molecules into the pores produces a redshift because the APTES molecules displace the air from the pores. The redshift of resonances can be easily detected by optical reflectance. As can be seen in Figure 7c, a redshift of 44.3 nm was obtained with respect to the oxidized sample (Figure 7b). Even by using low concentration of APTES (approximately 5%), the spectral displacements are larger than the previously reported ones in PSi structures that were infiltrated with several molecular species [44,45]. This effect can be attributed to the large specific surface area of the PSi that allows us to have a large quantity of available sites for chemical binding. This idea is based on the saturation curves obtained for APTES in micro- and mesoporous structures reported in reference [46] and confirmed in our samples (not shown for brevity). In order to compare the sensitivity of the APTES infiltration in HHs and the structure given in reference [45], we have calculated the *ΔE* (in electron volt) from the spectral position of the resonance modes before and after the APTES infiltration. In that reference, *ΔE*∼ 0.27 eV for structures designed at 830 nm. In our case, *ΔE*=0.053 eV for *λ*=1.0 *μ*m, according to the pore size criteria given above. An important characteristic that the HHs offer is the possibility to generate strong localized transmission modes that allow us to better monitor their spectral displacements even at a low concentration of analyte.

Theoretical simulation is also presented in Figure 7 (dashed line). In this case, unlike the first simulations and as a result of changes in the effective refractive index produced by thermal oxidation and APTES infiltration, we introduced a constant *Δn*_*i*_ in order to reproduce the best possible experimental reflectance, where *i* corresponds to layers *A*, *B*, *C* and *D*. The values of *Δn*_*i*_ were adjusted, taking into account that the silicon dioxide grows at the expense of silicon (reducing the effective refractive index) and APTES displaces the air from the pores (increasing the effective refractive index). We found that the values of *Δn*_*i*_ induced by the APTES attachment on the oxidized layers is ≤0.09 for each layer. Figure 7 shows a good agreement between the experimental and calculated spectra. The simulation of the optical spectra, based on experimental data of the HHs structures with the molecule infiltration, could give us a quantitative analysis method for material infiltration even at a very low concentration.

## Conclusions

In conclusion, we have been able to demonstrate the fabrication of hybrid heterostructures consisting of dielectric multilayers of distributed Bragg reflectors and Fibonacci type. Even considering the complexity of the HHs and the multiple factors involved in PSi formation, we obtain a very high quality and reproducibility in PSi multilayers in our experimental setup. The possibility to localize resonant modes and tuning them has been proven. Unfolding of resonant modes can be generated by repeating periodically the hybrid structure. The theoretical model is in good agreement with the experimental data, so it could be used to estimate changes in optical responses of chemically modified structures. Such hybrid heterostructures can be very promising in the field of optoelectronics, optical communications [47], and biosensors [48].

## Competing interests

The authors declare that they have no competing interests.

## Authors’ contribution

KP carried out all the experimental processes, optical characterization and drafted the manuscript. AM participated in performing the process of porous silicon, analyzed the characterization data and drafted the manuscript. JOE and JA developed the theoretical calculation programs. GP designed the infiltration protocol of biological molecules into porous silicon. MEM made the initial theoretical model of heterostructures based on porous silicon. All authors read and approved the final manuscript.
